# A closer look at the role of deubiquitinating enzymes in the Hypoxia Inducible Factor pathway

**DOI:** 10.1042/BST20230861

**Published:** 2024-11-25

**Authors:** Tekle Pauzaite, James A. Nathan

**Affiliations:** Cambridge Institute of Therapeutic Immunology & Infectious Disease (CITIID), Jeffrey Cheah, Biomedical Centre, Department of Medicine, University of Cambridge, Cambridge CB2 0AW, U.K.

**Keywords:** deubiquitination, DUB, hypoxia, hypoxia inducible factors, oxygen sensing

## Abstract

Hypoxia Inducible transcription Factors (HIFs) are central to the metazoan oxygen-sensing response. Under low oxygen conditions (hypoxia), HIFs are stabilised and govern an adaptive transcriptional programme to cope with prolonged oxygen starvation. However, when oxygen is present, HIFs are continuously degraded by the proteasome in a process involving prolyl hydroxylation and subsequent ubiquitination by the Von Hippel Lindau (VHL) E3 ligase. The essential nature of VHL in the HIF response is well established but the role of other enzymes involved in ubiquitination is less clear. Deubiquitinating enzymes (DUBs) counteract ubiquitination and provide an important regulatory aspect to many signalling pathways involving ubiquitination. In this review, we look at the complex network of ubiquitination and deubiquitination in controlling HIF signalling in normal and low oxygen tensions. We discuss the relative importance of DUBs in opposing VHL, and explore roles of DUBs more broadly in hypoxia, in both VHL and HIF independent contexts. We also consider the catalytic and non-catalytic roles of DUBs, and elaborate on the potential benefits and challenges of inhibiting these enzymes for therapeutic use.

## Introduction

The ability of organisms to sense and adapt to varying oxygen gradients is a highly conserved process. In metazoans, cellular oxygen levels are principally sensed by prolyl hydroxylases (PHDs, also known as EGLNs) that in turn control the effector transcriptional response through stabilisation of HIFs [1–3]. Under normal oxygen tensions, the HIF-α subunit is hydroxylated by PHDs at two conserved proline residues. This prolyl hydroxylation facilitates the recruitment of the Von Hippel Lindau (VHL) E3 ligase, leading to HIF-α ubiquitination and rapid proteasome-mediated degradation. In hypoxia, there is insufficient oxygen for PHD catalytic activity, therefore HIF-α is not ubiquitinated by VHL and can bind to its constitutively expressed HIF1β counterpart, forming an active dimeric transcription factor [4–7]. This complex translocates to the nucleus, binds hypoxia responsive elements at the HIF responsive genes, and drives a hypoxic adaptation programme [8–11].

There are three HIF-α isoforms (HIF-1α, HIF-2α and HIF-3α), with HIF-1α and HIF-2α having similar but also distinct roles in HIF transcriptional responses [8,10–19]. The role of HIF-3α in hypoxia is less clear and here we use HIF-α to refer to the common functions of the HIF-1α and HIF-2α isoforms. There are also three PHD forms (PHD1-3) [20–25]. Again, these have overlapping and distinct roles, but PHD2 seems to act as the dominant hydroxylase enzyme for HIF-α [26,27].

In this review, we explore the complex nature of ubiquitin-mediated regulation of the HIF response, and focus on the multifaceted roles of deubiquitinating enzymes (DUBs) in tuning this pathway. We start with the discovery that VHL-mediated ubiquitination provides the dynamic and sensitive nature of HIF signalling, before moving on to explore how ubiquitination of HIF can be regulated by DUBs. We also consider how the different properties of DUBs may influence HIF signalling. Lastly, we explore the role of DUBs in other aspects of hypoxia biology, and speculate on the potential future therapeutic applications of these enzymes.

## VHL-mediated ubiquitination is central to HIF regulation

Ubiquitination is well characterised as the major route for targeting proteins for degradation by the 26S proteasome [28,29], but ubiquitin also serves non-degradative roles in many cellular processes including cell signalling, chromatin regulation, and protein trafficking [30–34]. The ability of ubiquitin to regulate such diverse outcomes relates to its ability to form eight different linkage types with itself (through its 7 lysines or N-terminus), and modify proteins with different ubiquitin patterns (monoubiquitination, multiple monoubiquitination, homotypic polyubiquitin chains, heterotypic polyubiquitin chains, and branched linkages). Ubiquitin conjugation has also been recently identified on sugar and lipid moieties [35–37], and combining these with the diverse outcomes from protein ubiquitination has resulted in the ‘ubiquitin code’ providing arguably the most complex regulatory post-translational modification within cells.

Ubiquitination by VHL provided one of the earliest examples of rapid protein degradation regulating a transcriptional response. It may at first seem counterintuitive to control a transcriptional pathway through protein degradation, rather than transcription or translation [38–42]. However, regulation of proteolysis allows the rapid initiation and termination of the HIF response, with HIF-α having a half-life of <5 min under oxygenated conditions [22,38,43–49]. The dominant nature of VHL within the HIF pathway is exemplified by human *VHL* germline mutations (VHL disease) that exhibit HIF stabilisation and expression of HIF target genes [50,51]. These vascularised tumours, which include haemangioblastomas of the retina and central nervous system, clear cell renal carcinomas (ccRCCs) and pheochromocytomas, all express high levels of vascular endothelial growth factor and erythropoietin, which are well validated targets of HIFs [52–54]. The dominant nature of VHL within HIF signalling is also exemplified by somatic loss of VHL driving the initiation of sporadic ccRCCs. Around 85% of ccRCCs arise from loss of VHL function, which is typically associated with constitutive HIF-2α stabilisation [55–57]. HIF-2α depletion and inhibition suppresses VHL null ccRCC progression [58–60], and HIF-2 inhibitors are now in clinical use for advanced-stage ccRCC and VHL disease [61,62].

While prolyl hydroxylation and VHL-mediated ubiquitination provides the dominant mechanism for turning HIF-mediated transcription on, how HIFs are regulated under hypoxic conditions, or turned off, may involve other regulatory processes. HIF-1 and HIF-2 complexes demonstrate different stabilisation kinetics under hypoxia, and this may be partly explained by transcriptional and translational control of HIFs [63–67]. Additionally, downstream activation of HIF target genes can be regulated by the rate of the HIF heterodimer complex translocation to nucleus, HIF chromatin binding, epigenetic modifications at HIF target genes, and through selective gene activation by the different HIF isoforms [22,68–71]. A number of HIF-α post-translational modifications aside from hydroxylation may also be important for fine-tuning the HIF response, including acetylation [72–74], phosphorylation [75], SUMOylation [76–78], VHL-independent ubiquitination [79–90], and deubiquitination [70,90–98].

## Does deubiquitination counteract the functions of VHL?

Approximately 100 DUBs have been identified in humans to date that are classified into seven families: Ub C-terminal hydrolases (UCHs), Ub-specific proteases (USPs), Machado-Josephin domain proteases, ovarian tumour proteases (OTUs), motif interacting with Ub-containing novel DUB family (MINDY), and zinc-finger-containing Ub peptidase (ZUP1) are all cysteine based proteases; and DUBs that belong to the Jab1/Mov34/MPN+ protease (JAMM) family are zinc-binding metalloproteases [99–101]. The general function of deubiquitinases is to maintain ubiquitin homeostasis and it is achieved by four different modes of action: (1) processing, maturation, and release of the free ubiquitin [102]; (2) removal of ubiquitin chains, rescuing proteins from degradation or modifying signalling pathways; (3) editing of ubiquitin chains that can change ubiquitin signal [103–105]; and (4) recycling of ubiquitin by proteasome-associated DUBs that both activate the 26S and ensure that ubiquitin is not degraded together with the substrate [106–109].

Given that the major regulator of HIF signalling is VHL, the obvious question is whether deubiquitinases play a significant role in HIF control of VHL activity? This could occur through regulation of VHL itself, controlling PHD stability, or through reversing HIF-α ubiquitination.

**Figure 1.**
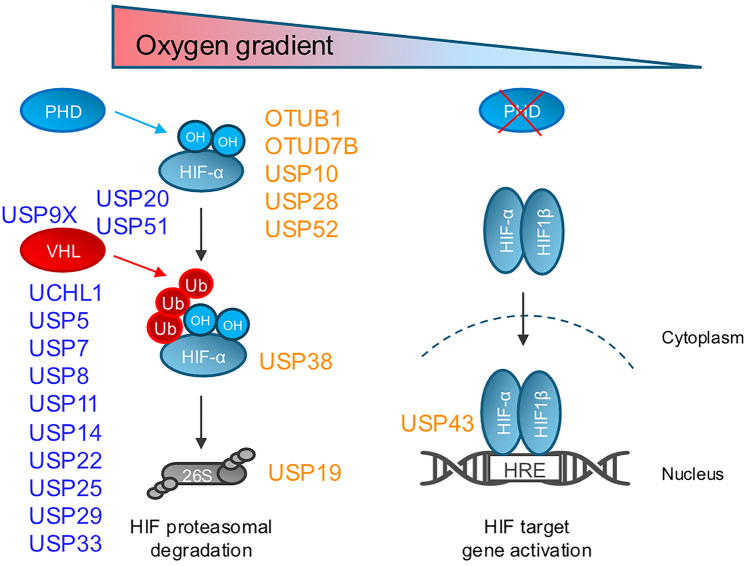
Deubiquitinating enzymes involved in HIF signalling. Schematic illustrating the HIF pathway in high or low oxygen levels, and its potential regulation by DUBs. DUBs that regulate VHL or alter HIF-α stability (blue). DUBs that act in a VHL independent manner and regulate the HIF response (orange). HRE, hypoxia responsive element; 26S, proteasome.

Several DUBs have been linked to regulation of the VHL-PHD axis, including both USP9X and USP19. USP9X has been reported to counteract SMURF1-mediated ubiquitination of VHL [110] (Figure 1, Table 1), but as USP9X has multiple targets it is unclear how important the regulation of VHL is. USP19 interacts with the SIAH1/2 ubiquitin E3 ligases that can affect the stability of PHDs [89,126–128].

**Table 1.** DUBs that have been linked with VHL-dependent regulation of HIFs by deubiquitination

DUB	Effect on HIF-α	Biological effects	Changes in DUB levels in hypoxia	Cell types/models	References
**HIF response suppressors**					
USP9X	Reduces VHL levels and increases HIF-1α stability	Activates glycolysis and promotes cell proliferation	NK	786-O, HEK-293T, HepG2, PC3, B16, HCT116	[110]
**HIF response activators**					
UCHL1	Counteracts VHL mediated ubiquitination of HIF-1α	Promotes pulmonary metastasis	Increased in hypoxia	Murine models of EMT6 and B16F10 pulmonary metastasis	[91,111–113]
USP5	Interacts with and stabilises HIF-2α	Promotes proliferation, colony formation, migration, and invasion	Protein levels increased in hypoxia	Human breast cancer cell lines: MCF-7, MDA-MB-231	[114]
USP7	Deubiquitination and stabilisation of HIF-1α	Promotes epithelial-mesenchymal transition and metastasis	Protein levels increased but mRNA reduced in hypoxia	HEK-293T, H1299, *in vivo* H1299 EMT and metastatic murine models, mouse skin fibroblasts	[94,115]
USP8	Deubiquitinates and stabilises HIF-1α and HIF-2α	Primary cilia formation	Reduced in hypoxia	MEFs, RPE-1, RPTEC	[116]
USP11	Deubiquitinates and stabilises HIF-1α	Increase glycolysis	mRNA reduced in hypoxia	Human HCCs: PLC/PRF/5, Hep3B, HEK-293T and Huh7	[117]
USP14	Stabilisation of HIF-1α by deubiquitination	Promotes tumour growth, cell migration, invasion	Protein levels reduced in hypoxia	HCCs: HCCLM3, Huh-7, immortalised hepatocyte cell lines, murine tumour transplantation models	[118]
USP20 - VDU2	Interacts with VHL, stabilises HIF-1α	Increase sexpression of HIF-1 target genes	NK	COS-7, HeLa and 786-O	[119]
USP22	Deubiquitinates and stabilises HIF-1α	Promotes TP53 deficient HCC stemness and glycolysis	mRNA is increased in hypoxia	HCCs: BEL-7402, Huh-7, PLC/PRF/5 and SK-Hep-1	[120]
USP25	Deubiquitinates and stabilizes HIF-1α, USP25 depletion increases HIF-1α mRNA levels	Metabolic reprogramming and survival in PDAC	NK	Murine derived pancreatic ductal adenocarcinoma (PDAC), PDAC organoids, and patient-derived organoids, *in vivo* subcutaneous transplantation PDAC model	[121]
USP29	Deubiquitinates and stabilizes HIF1α under normoxic conditions	Promotes glycolysis, drives resistance to therapy with the multi-kinase inhibitor Sorafenib	NK	HCCs: SNU398 Huh7, HLE, Hep3B; murine tumour transplantation models	[122]
USP29	Directly deubiquitinates and stabilizes HIF1α and MYC	Drives biosynthesis and tumour survival	NK	TH-MYCN neuroblastoma and Eμ-Myc B cell lymphoma mice models, SK-N-BE2, Ramos, A549 and HEK-293T	[123]
USP33	ERK1/2 dependent deubiquination and stabilisation of HIF-2α	Glioma stem cells maintenance, tumour vascularization, and growth	mRNA and protein levels increased in hypoxia	Human glioblastoma specimens, glioma stem cells, and non-stem tumour cells	[124]
USP51	Fine-tunes VHL activity by associating with HIF-1α through Elongin C — member of VHL E3 ligase complex	Proliferation, migration, stemness, and chemoresistance under hypoxia	mRNA increased by HIF-α driven transcription	Colorectal cancer cell lines: HCT116, DLD-1, SW480, and LoVo	[125]

The effect on HIF protein or transcription, the phenotypic outcome, DUB regulation by hypoxia, and the cell lines/models used are listed. NK, not known.

While VHL or PHD levels may be controlled to some extent by ubiquitination, many more DUBs have been proposed to oppose or fine-tune VHL mediated ubiquitination of HIF (Figure 1, Table 1). For example, USP51 and USP20 have been reported to directly associate with VHL, enabling them to oppose VHL mediated HIF-α ubiquitination more efficiently [119,125]. USP51 associates with VHL through the Elongin C subunit and counteracts HIF-1α ubiquitination [125]. USP51 is itself a direct target of HIF-1α and HIF-2α driven transcription, which suggests that there is a positive feedback loop for control.

Many of the DUBs linked to HIF-α deubiquitination seem to show cell line or cancer type specificity (Figure 1, Table 1). Limitations of some of these studies relate to a reliance on overexpression of the DUBs. Nonetheless, there is a growing body of research suggesting that DUBs can oppose VHL directly in specific contexts. USP11, USP22, and USP29 have been shown to deubiquitinate and stabilise HIF-1α in human HCC cells [117,122,123]. Hypoxia also alters the expression of these DUBs in hepatocellular cells, with USP11 being down-regulated but conversely USP22 is up-regulated [117,120]. USP33 deubiquitinates and stabilises HIF-2α in glioma cells, aiding glioma stem cell maintenance, tumour vascularization, and glioblastoma growth [124]. USP25 has been suggested to directly counteract VHL by deubiquitination and stabilisation of HIF-1α in pancreatic ductal adenocarcinoma models [121]. Interestingly, even though USP25 depletion increases HIF-1α transcription, its effect on HIF driven gene transcription is still detrimental. USP5 has been shown to interact with and deubiquitinate HIF-2α in breast cancer models, and its protein level is positively correlated with HIF-2α protein levels in human breast cancer tissues [114]. UCHL1 promotes pulmonary metastasis in murine models by abrogating VHL-mediated ubiquitination of HIF-1α [111,112,129,130]. USP7 has been proposed to deubiquitinate and stabilise HIF-1α in several cancer line cell types [115]. Finally, USP8 has been reported to deubiquitinate both HIF-α isoforms in non-cancer cell lines (293T, mouse embryonic fibroblasts (MEFs) and RPE-1 cells), with a reported role in primary cilia formation [116].

Together, while these studies indicate some involvement of DUBs in tuning HIF-α levels, VHL is still dominant in regulating HIF stability. It is also noteworthy that DUBs may have pleotropic effects through deubiquitinating multiple targets. For instance, the predominant effects of USP7 inhibition in cancers relates to MDM2 destabilisation and p53 stabilisation, and not HIF regulation [131].

## VHL-independent regulation of HIFs by deubiquitination

While counteracting VHL activity is unlikely to be the main function of DUBs within the HIF pathway, deubiquitination may be involved in other aspects of the HIF response, including transcription of the HIF-α isoforms, HIF localisation, nuclear translocation, or the control of protein synthesis. To interrogate the functions of DUBs in an unbiased manner, we recently undertook a functional genomics approach to find DUBs that regulate HIF-α stability or modulate a HIF-mediated transcriptional response in cancer and non-cancer cell lines [70]. Interestingly, in these CRISPR mutagenesis screens, DUBs that regulated HIF-α stability were not observed, reflecting redundancy or that VHL remains dominant in the conditions tested. However, two DUBs were identified with high confidence as being required for activating the HIF response, namely USP43 and USP52 (PAN2). USP52 is a pseudo-DUB that was previously identified to stabilise HIF-1α mRNA at the P bodies, therefore potentiating the HIF transcriptional response [98]. USP43 is less studied, but we and others have shown that it co-operates with 14-3-3 adaptor proteins independently of its catalytic activity [70,132,133]. This interaction with 14-3-3 proteins is required for activation of a HIF-1 but not HIF-2 response, facilitating nuclear accumulation of the HIF-1 complex and activation of HIF-1 target genes [70]. USP43 is itself up-regulated in hypoxia in a HIF-dependent manner, demonstrating a forward feedback loop that is commonly observed with HIF target genes [70]. Whether there is an additional function of USP43 catalytic activity in HIF signalling remains to be seen, but similarly to USP52 and USP19, it is important to consider that non-catalytic functions of DUBs can have functional consequences [70,95,98] (Figure 1, Table 2).

**Table 2.** DUBs that have been linked with VHL-independent regulation of HIFs by deubiquitination

DUB	Effect on HIF-α	Biological effects	Changes in DUB levels in hypoxia	Cell types/models	References
**HIF response suppressors**					
USP10	Regulates mTOR/S6K mediated HIF-1α synthesis	Glycolysis, cell proliferation, migration, and adhesion	mRNA and protein reduced in hypoxia	HEK-293T, MEFs, HCT116, COLO320	[94]
**HIF response activators**					
OTUB1	Non-canonical inhibition of HIF-1α ubiquitination and HIF-1α stabilisation, independent from PHD/VHL and FIH	Promotes hypoxia-induced glycolytic reprogramming for cellular metabolic adaptation	mRNA increased in hypoxia	HEK-293T and H1299	[91]
OTUB1	Covalently binds FIH in hypoxia	Regulate energy expenditure, age-dependent body weight gain, blood glucose clearance and plasma insulin levels in mice	Hypoxia increases OTUB1 enzymatic activity	HEK-293T, MCF7, Hep3B; MEFs and murine models; MDA-MB468, HepG2, Hep3B, Caco-2, A549; murine tissues and organs	[134-137,138]
OTUD7B (Cezanne)	Regulates HIF-2α expression in E2F1- dependent manner, stabilises E2F1 for HIF-2α transcription	HIF-2α homeostasis	NK	HeLa and HEK-293T, 786-O, murine tissues and organs	[93]
OTUD7B (Cezanne)	Regulates HIF-1α protein levels at post-translational level, via targeting HIF-1α for lysosomal degradation	HIF-1α homeostasis	mRNA increased in endothelial cells, but not in U2OS	U2OS, HeLa, HEK-293T, RCC4, A498, MEFs	[92]
USP19	Stabilises HIF-1α independently of its catalytic activity, potentially by rescuing it from proteasomal degradation	Activates glycolysis and angiogenesis	NK	HeLa, HEK-293T, human melanoma cell line M2, U2OS	[95]
USP28	Prevents FBXW7 dependent HIF-1α degradation during hypoxia	Increases transcriptional activity of HIF-1α in cells	mRNA and protein reduced in hypoxia	HCT116, HepG2, HeLa, and HEK-293T, HMEC-1, MEFs	[90,96,139]
USP38	Deubiquitinates and stabilises HIF-1α independently from PHD/VHL	Potentiates hypoxic response	NK	H1299 cell line	[97]
USP43	Aids HIF-1α binding to chromatin	Positively regulates HIF-1α transcriptional response	mRNA and protein increased in hypoxia	HEK-293T, HeLa, A549, MCF-7, HKC-8, RPE-1, 786-O, RCC4	[70]
USP52 (PAN2)	Stabilises HIF-1α mRNA at the P bodies	Potentiates hypoxic response	NK	U2OS, HeLa, HEK-293T, RCC4, 786-O	[98]

The effect on HIF protein or transcription, the phenotypic outcome, DUB regulation by hypoxia, and the cell lines/ models used are listed. NK, not known.

Other VHL-independent roles for DUBs in HIF signalling have been explored in relation to non-canonical HIF ubiquitination. There remains controversy regarding the importance of VHL-independent HIF-α ubiquitination, but it is worth noting that DUBs have been implicated in these pathways (Figure 1, Table 2). For instance, USP38 interacts with HIF-1α to deubiquitinate K11-linked polyubiquitination of HIF-1α at Lys769, stabilising HIF independently from the PHD/VHL axis [97]. OTUB1 is responsible for non-canonical inhibition of HIF-1α ubiquitination and HIF-1α stabilisation that is independent from PHD/VHL and Factor Inhibiting HIF (FIH) [91]. OTUB1 has also been extensively studied in relation to FIH, where it can covalently bind FIH in hypoxia and regulate energy expenditure, age-dependent body weight gain, blood glucose clearance and basal plasma insulin levels in mice [134–137]. USP28 is an additional DUB that may regulate HIF-α ubiquitination independently of VHL. USP28 counteracts FBXW7 dependent ubiquitination and HIF-1α degradation during hypoxia. In turn, the stabilisation of HIF-1α in hypoxia leads to activation of USP28 through SENP1-mediated USP28 deSUMOylation to further increase transcriptional activity of HIF-1 in human cancer cell lines [90,96].

USP10 and OTUD7B (Cezanne) provide interesting examples of DUBs implicated in regulating HIF-α at the transcriptional and translational level (Figure 1, Table 2). USP10 depletion increases mTOR/S6K mediated HIF-1α but not HIF-2α protein synthesis in normoxic and hypoxic conditions in colon cancer cell lines. Cezanne confers different activities towards specific isoforms of HIF. It regulates HIF-2α expression in E2F1- dependent manner by stabilising E2F1 for HIF-2α transcription [93]. Additionally, Cezanne regulates HIF-1α protein levels at the post-translational level, via chaperone-mediated autophagy [92].

## The involvement of DUBs in HIF-independent oxygen-sensitive responses

It is also worth considering the potential involvement of DUBs in other cellular responses to oxygen availability. Whilst VHL and HIFs are central to metazoan responses to hypoxia, other oxygen-sensing pathways can occur and VHL can ubiquitinate other proteins aside from HIF-α. For example, USP13 has been reported to be induced in hypoxia and deubiquitinate toll-like receptor 4 (TLR4), leading to nuclear factor-κB (NF-κB) activation [140]. Interestingly, USP13 has also been implicated in renal cancer, but rather than altering HIF activity, USP13 deubiquitinates another VHL target gene and oncogenic driver, ZHX2 [141].

Several DUBs are involved in ischaemia reperfusion injury (IRI). USP29 deubiquitnates TGF-β activated kinase 1 (TAK1) and alleviates ischaemic liver injury by reducing inflammation and apoptosis [142]. Similarly, USP10 has been suggested to protect against hepatic IRI via TAK1 signalling [143]. OTUD4 and Cezanne remove K63-linked polyubiquitin chains on tumour necrosis factor receptor-associated factor 6 (TRAF6), alleviating inflammation and IRI in the liver and kidney respectively [144,145], and USP8 alleviates intermittent hypoxia/reoxygenation induced inflammation by removing K63-linked ubiquitination of TAK1 [146]. In contrast, USP11 aggravates IRI by promoting the deubiquitination of 3 (TRAF3) [147].

DUBs have also been implicated in recovery after cerebral hypoxic damage, such as USP14 in neonatal hypoxia-ischaemia encephalopathy, and UCHL1 which ameliorates autophagy and tissue damage after traumatic brain injury [129,148].

Lastly, an area closely linked to hypoxia biology is radiosensitivity, and DUBs are potentially important in altering cell sensitivity to ionising radiation due to their involvement in DNA damage repair, the cell cycle, and cell death. Generating small molecule DUB inhibitors or novel deubiquitinase-targeting chimeras [149] is an attractive strategy for radiosensitisation. The most prominent ubiquitin enzymes in hypoxia radiosensitivity regulation are the E3 ligases Parkin, VHL, and TRIM21; and the DUBs USP20, USP25, USP28, and UCHL1 (reviewed in [150,151]).

## Limitations of current DUB research in hypoxia and HIF biology

Research on DUBs in hypoxia is growing rapidly, but one of the main challenges to gain a comprehensive understanding is the apparent cell specificity of some of the findings. Many prior studies have relied on overexpression systems [113,115,116,119], and there are few *in vitro* studies confirming direct action of DUBs on VHL-mediated ubiquitination of HIF-α. There are technical challenges in undertaking these studies, as DUBs are typically large proteins, have multiple domains, can work in complexes, and have pleotropic effects. When unbiased functional genomic approaches have been used [70,98,121], they have not detected DUBs that counteract VHL-mediated ubiquitination of HIF-α. These findings point to VHL being the dominant regulator of HIF stability, as supported by genetic mutations altering VHL function [152,153]. This makes the discovery of DUBs opposing VHL unlikely to have a biological relevance *in vivo*. Why some DUBs act in specific cell types or cancer models is not clear, and future studies will need to move to models more representative of primary cells and patient-derived tumours. Further developments in DUB structural biology, enzymology and the generation of selective DUB inhibitors will provide key tools to explore the roles of DUBs in hypoxia, without the reliance on overexpression or sustained depletion of DUBs, thereby avoiding non-specific or compensatory effects.

## Therapeutic perspectives

Pharmacological manipulation of HIFs has already shown promise in clinical settings. Several PHD inhibitors are at various stages of approval or treatment for anaemia due to chronic kidney disease [154–161]. The selective HIF-2 inhibitor, Belzutifan (PT2977), is licenced for treatment of advanced-stage ccRCC and VHL disease [61,162,163]. However, the acquired resistance to HIF-2α inhibition [163,164] is likely to need combined therapeutic strategies, where DUB inhibitors could play a role. Additionally, as some DUBs show specificity to the different HIF isoforms (Tables 1 and 2), DUB inhibitors may help target different aspects of the HIF response, but important questions remain regarding the disease settings where they may be useful.

There are several DUB inhibitors in preclinical studies, including USP1, USP8, USP9X, USP10, USP13, USP14, USP47, and UCHL1 inhibitors [109,165–172]; and a few in clinical trials (e.g. the USP30 inhibitor, MTX652, and a USP1 inhibitor, KSQ-4279) [173,174]. However, there are reasons why finding selective and efficient DUB inhibitors is complicated. Technical challenges in drug design and DUB biochemistry combined with the broad functions of DUBs have been a limitation in the field. Furthermore, DUBs can have both catalytic and catalytic-independent roles [70], and where catalysis is important, DUBs have a relatively conserved catalytic site structure that leads to a lack of potent inhibitor selectivity [175]. Finally, many DUBs undergo allosteric regulation required for conformational changes, localisation and activation [176–179]. Despite these challenges, we should be encouraged by the serendipitous success of other inhibitors targeting components of the ubiquitin-proteasome system in disease, such as proteasome inhibitors. Targeting the main machinery for selective protein breakdown would at first glance seem unlikely to be of therapeutic value, but observations of selective cell death in plasma cells initiated the studies of proteasome inhibitors in myeloma, with this class of drugs becoming central to the treatment of this cancer [180]. DUB inhibition therefore still holds potential therapeutic promise, and only by understanding the cellular contexts in which DUBs function can we determine where their therapeutic potential may lie.

## Open Access

Open access for this article was enabled by the participation of University of Cambridge in an all-inclusive *Read & Publish* agreement with Portland Press and the Biochemical Society under a transformative agreement with JISC.

## Abbreviations

DUB: deubiquitinating enzyme
FIH: Factor Inhibiting HIF
HIF: Hypoxia Inducible transcription Factor
IRI: ischaemia reperfusion injury
MEF: mouse embryonic fibroblast
OTU: ovarian tumour protease
PDAC: pancreatic ductal adenocarcinoma
PHD: prolyl hydroxylases
TAK1: TGF-β activated kinase 1
UCH: Ub C-terminal hydrolase
USP: Ub-specific protease
VHL: Von Hippel Lindau
